# Supplementary material for: Generalized precursor prediction boosts identification rates and accuracy in mass spectrometry based proteomics
Source: Commun Biol. 2023 Jun 10;6:628. doi: 10.1038/s42003-023-04977-x (PMC10257694; doi:10.1038/s42003-023-04977-x)
Supplement: Supplementary file 2 — Supplementary Figures [file 42003_2023_4977_MOESM2_ESM.pdf]

# Generalized precursor prediction boosts identification rates and accuracy in mass spectrometry based proteomics

Aaron M. Scott[1], Christofer Karlsson[1], Tirthankar Mohanty[1], Erik Hartman[1], Suvi T. Vaara[2], Adam Linder[1], Johan Malmström[1], and Lars Malmström[1]

[1]Division of Infection Medicine, Department of Clinical Sciences, Lund University, Lund, Sweden
[2]Division of Anaesthesia and Intensive Care Medicine Department of Surgery, Intensive Care Units, Helsinki University Central Hospital, Box 340, 00029 HUS, Helsinki, Finland

# 1 Supplementary Figures

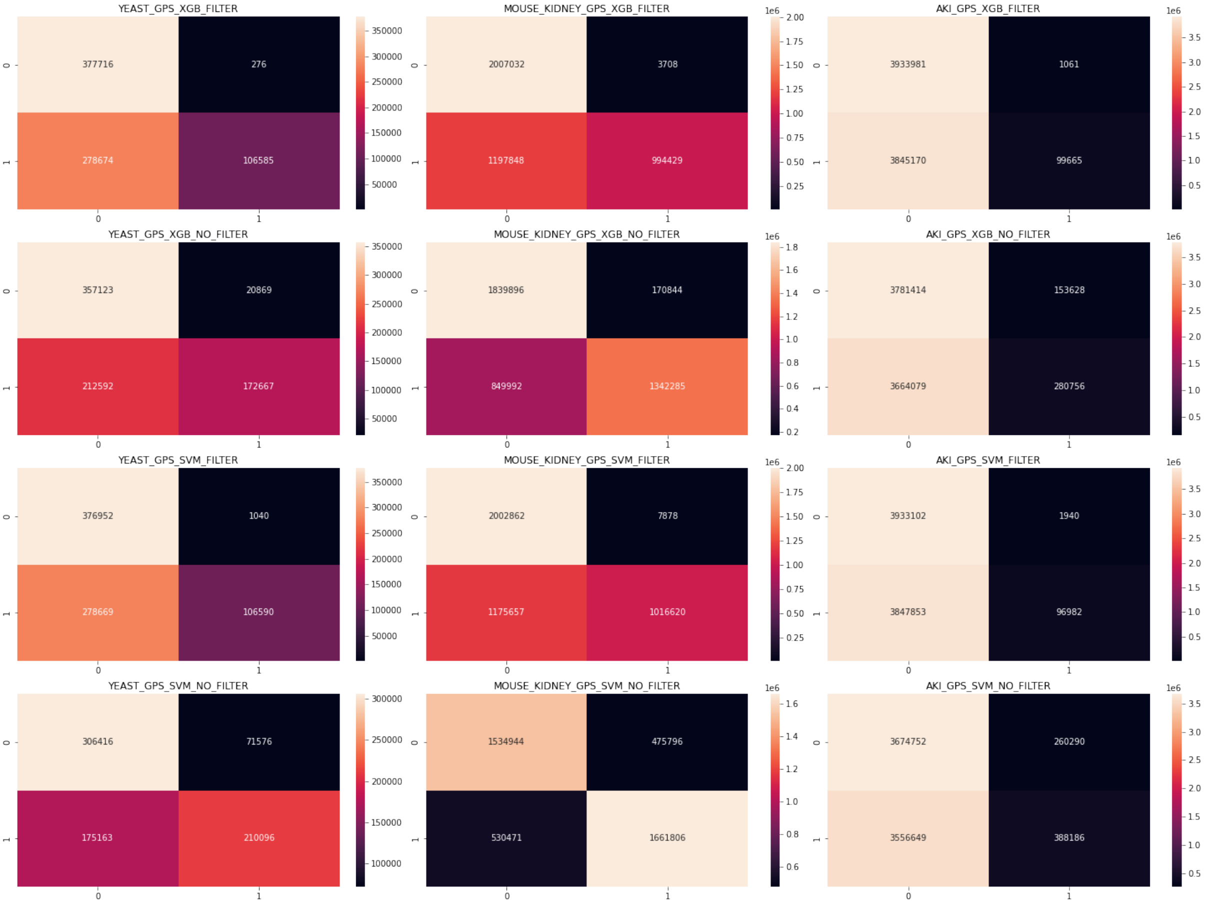


**Supplementary Figure S1: Confusion matrices for all GPS models trained for the filtered and unfiltered data sets.** These confusion matrices display the true target (1) and decoy (0) predictions for each of the 4 trained classifiers on each of the 3 test datasets. The 2 models trained on unfiltered data (No Filter) in rows 2 and 4 display a substantially greater number of false positive predictions (ie. decoys predicted as targets) compared to the Filter models.

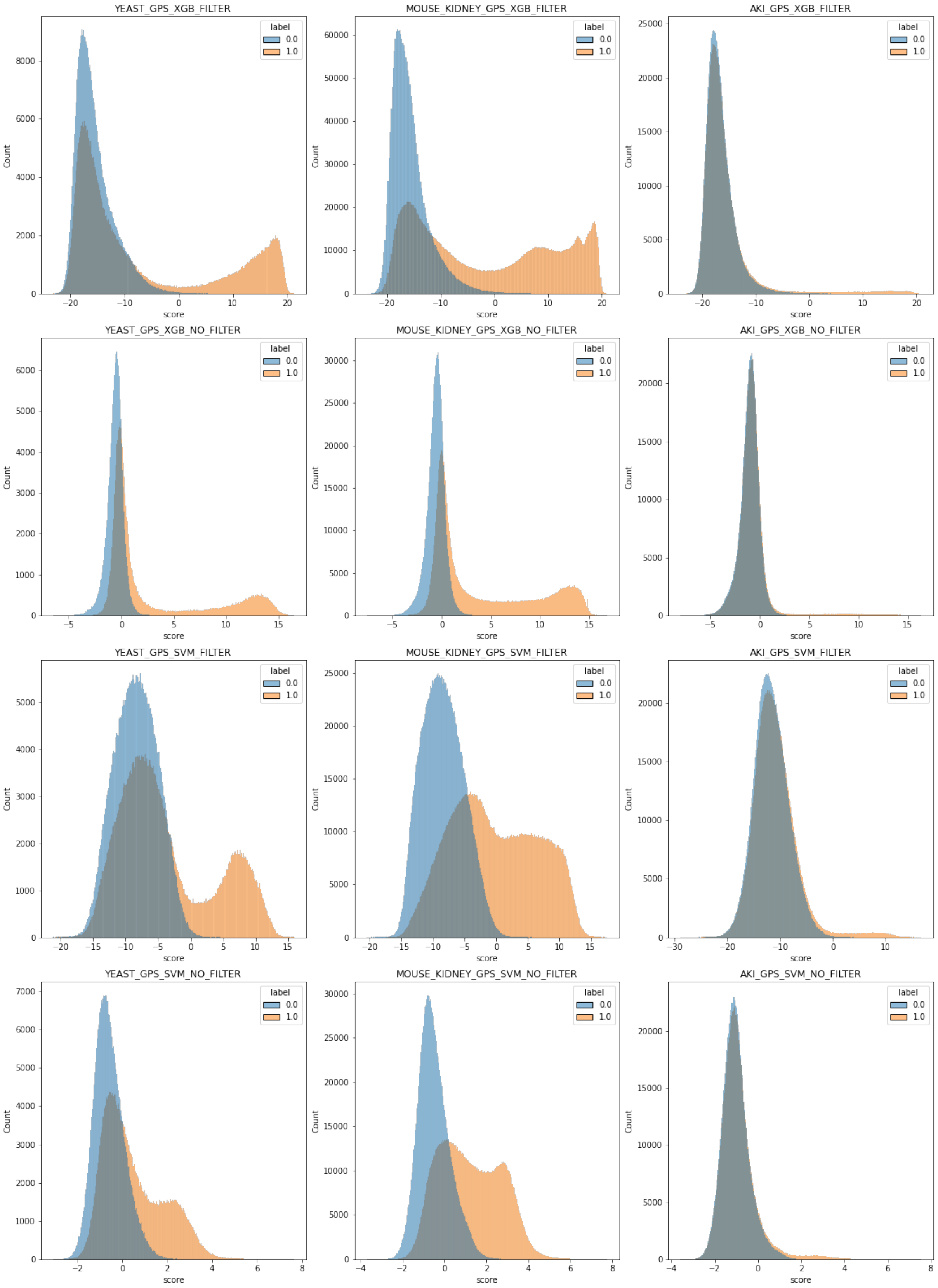


**Supplementary Figure S2: Scoring distributions for all GPS models trained for the filtered and unfiltered data sets.** These scoring distributions display the target (1.0) and decoy (0.0) scores for each of the 4 trained classifiers on each of the 3 test data sets. The XGBoost models in the first 2 rows show better separation of the decoy and target classes than the SVM models in the bottom 2 rows. The ”false” target portions of the bimodal target distributions for the XGBoost models also more closely match the decoy distributions than the SVM models, indiciation that q-value calculation and FDR control should be more accurate.

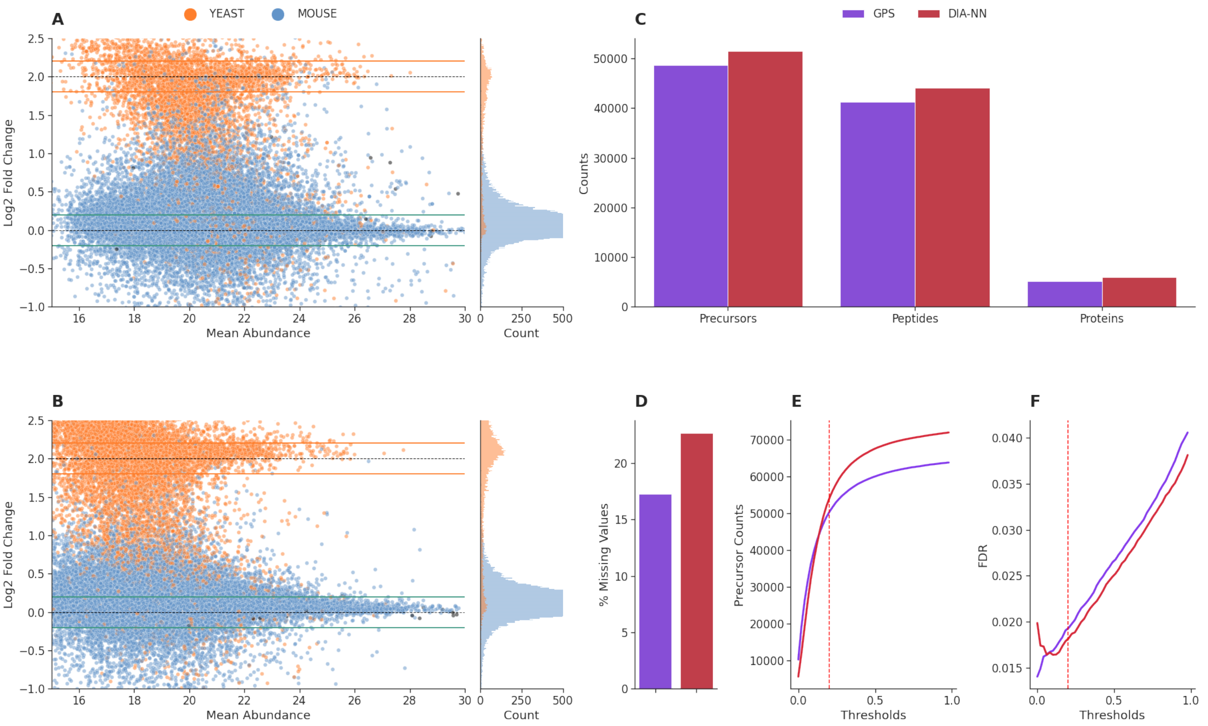


**Supplementary Figure S3: Quantification accuracy of GPS evaluated by a 2 species mixture spike-in data set compared to DIA-NN.** We evaluated the quantification accuracy of GPS by analyzing a 2-species mixture of yeast peptides spiked-in into a constant mouse kidney proteome background with 2 groups of 10 technical replicates each. Each group of samples contained the same concentration of Mouse-Kidney proteins, while one group contained 4X more yeast peptides and we measured the number of precursors that mapped correctly into the expected ratio of their species (0.0 $\pm$0.2 log2 fold change for Mouse precursors and 2.0 $\pm$0.2 log2 fold change for Yeast precursors. **A)** displays the mean abundance of precursors identified using GPS against their log2 fold change and colored by their mapped species. Histogram plots directly to the right of these scatter plots display the distribution of the species mixture on the log2 fold change scale. The expected ratio regions are highlighted to display which precursors were considered as ratio-validated. **B)** displays the same as **A** but for DIA-NN. **C)** displays the overall counts of ratio-validated precursors, peptides, and proteins, from the regions highlighted in **A-B** for GPS and DIA-NN. From these validated regions, DIA-NN identifies more precursors, peptides, and proteins than GPS, mostly in the lower abundance regions. **D)** shows the percentage of missingness in the quantitative matrices for GPS and DIA-NN. Here, GPS decreased the number of missing values compared to DIA-NN. This is important in context with **C**, as GPS is able to provide a greater number of accurately quantified precursors and a substantially more complete data matrix as measured by the % missing values. In order to provide an evaluation beyond the ratio-validated cutoff, we measured the number of identified precursors and the FDR at increasing log2 fold change thresholds from the expected ratios of the species mixture (**E-F**). **E)** displays the number of precursors identified and quantified at increasing thresholds from the expected values. DIA-NN identifies more precursors at the 0.2 threshold, but less than GPS at thresholds closer to the expected rations. **F)** displays the the FDR as a function of increasing thresholds from the expected ratios of each proteome in the mixture. Here, we can see at low thresholds, DIA-NN displays a higher FDR, but the two tools even out over the measured thresholds, with DIA-NN having a lower FDR further away from the expected ratios.
