# Supplementary material for: Generalized precursor prediction boosts identification rates and accuracy in mass spectrometry based proteomics
Source: Commun Biol. 2023 Jun 10;6:628. doi: 10.1038/s42003-023-04977-x (PMC10257694; doi:10.1038/s42003-023-04977-x)
Supplement: Supplementary file 3 — Description of Additional Supplementary Files [file 42003_2023_4977_MOESM3_ESM.pdf]

Description of Additional Supplementary Files

File name: Supplementary Data 1

Description: The source data behind the graphs in the paper.

File name: Supplementary Data 2

Description: The trained GPS models used in the model comparison benchmark and throughout the rest of the study.
